# Epigenetic regulation of placental gene expression in transcriptional subtypes of preeclampsia

**DOI:** 10.1186/s13148-018-0463-6

**Published:** 2018-03-02

**Authors:** Katherine Leavey, Samantha L. Wilson, Shannon A. Bainbridge, Wendy P. Robinson, Brian J. Cox

**Affiliations:** 1 Department of Physiology, University of Toronto, 1 King’s College Circle, Toronto, ON Canada; 2 BC Children’s Hospital Research Institute, 950 W 28th Ave, Vancouver, BC Canada; 3 Department of Medical Genetics, University of British Columbia, C201-4500 Oak St, Vancouver, BC Canada; 4 Interdisciplinary School of Health Sciences, University of Ottawa, 25 University Private, Ottawa, ON Canada; 5 Department of Cellular and Molecular Medicine, University of Ottawa, 451 Smyth Rd, Ottawa, ON Canada; 6 Department of Obstetrics and Gynecology, University of Toronto, 23 Edward Street, Toronto, ON Canada

**Keywords:** Preeclampsia, Placenta, DNA methylation, Gene expression, Clustering, Subtypes

## Abstract

**Background:**

Preeclampsia (PE) is a heterogeneous, hypertensive disorder of pregnancy, with no robust biomarkers or effective treatments. We hypothesized that this heterogeneity is due to the existence of multiple subtypes of PE and, in support of this hypothesis, we recently identified five clusters of placentas within a large gene expression microarray dataset (*N* = 330), of which four (clusters 1, 2, 3, and 5) contained a substantial number of PE samples. However, while transcriptional analysis of placentas can subtype patients, we propose that the addition of epigenetic information could discern gene regulatory mechanisms behind the distinct PE pathologies, as well as identify clinically useful potential biomarkers.

**Results:**

We subjected 48 of our samples from transcriptional clusters 1, 2, 3, and 5 to Infinium HumanMethylation450 arrays. Samples belonging to transcriptional clusters 1–3 still showed visible relationships to each other by methylation, but cluster 5, with known chromosomal abnormalities, no longer formed a cohesive group. Within transcriptional clusters 2 and 3, controlling for fetal sex and gestational age in the identification of differentially methylated sites, compared to the healthier cluster 1, dramatically reduced the number of significant sites, but increased the percentage that demonstrated a strong linear correlation with gene expression (from 5% and 2% to 9% and 8%, respectively). Locations exhibiting a positive relationship between methylation and gene expression were most frequently found in CpG open sea enhancer regions within the gene body, while those with a significant negative correlation were often annotated to the promoter in a CpG shore region. Integrated transcriptome and epigenome analysis revealed modifications in TGF-beta signaling, cell adhesion, oxidative phosphorylation, and metabolism pathways in cluster 2 placentas, and aberrations in antigen presentation, allograft rejection, and cytokine-cytokine receptor interaction in cluster 3 samples.

**Conclusions:**

Overall, we have established DNA methylation alterations underlying a portion of the transcriptional development of “canonical” PE in cluster 2 and “immunological” PE in cluster 3. However, a significant number of the observed methylation changes were not associated with corresponding changes in gene expression, and vice versa, indicating that alternate methods of gene regulation will need to be explored to fully comprehend these PE subtypes.

**Electronic supplementary material:**

The online version of this article (10.1186/s13148-018-0463-6) contains supplementary material, which is available to authorized users.

## Background

Preeclampsia (PE) is a complex, heterogeneous disorder of pregnancy, diagnosed by the onset of maternal hypertension after the 20th week of gestation, with signs of maternal multi-organ dysfunction [1]. As with many pathologies of pregnancy, PE has no cure, robust predictive biomarkers, or effective treatments, other than the delivery of the infant to discontinue the pregnancy and remove what is thought to be the causative organ, the placenta. Repeated attempts to characterize the placental molecular pathology and identify biomarkers of PE by applying a binary approach (PE versus control) have not been clinically fruitful, and we hypothesized that this is due to the existence of multiple molecular subtypes of PE [2].

In support of this hypothesis, we recently published a large unsupervised clustering analysis of microarray data from a PE-focused placental cohort (*N* = 330), including 157 highly annotated samples purchased from a single biobank [3]. This revealed five clusters of placental gene expression containing at least three clinically significant etiological subtypes of PE: “maternal”, with term and near-term deliveries of average-sized infants and placentas that appear molecularly similar to normal healthy control samples; “canonical” with high placental expression of known PE markers, preterm deliveries, low fetal weights, and evidence of maternal malperfusion; and “immunological” with severe fetal growth restriction, enrichment of immune response genes, and histological signs of maternal anti-fetal/placental rejection [3], belonging to transcriptional clusters 1, 2, and 3, respectively. An additional subtype of PE placentas with chromosomal abnormalities was also discovered within cluster 5 (and supported by array-based comparative genomic hybridization (aCGH) analysis), but showed no strong clinical association [3].

However, despite our considerable progress towards understanding the molecular diversity observed amongst PE patients, RNA is relatively unstable, easily affected by technical variability [4], and rarely successful as a therapeutic target [5], limiting its clinical utility. We, therefore, propose that the integration of an additional level of molecular information in these placentas, such as DNA methylation, will compensate for these restrictions [4], as well as improve our understanding of the molecular pathology.

DNA methylation is a mitotically heritable epigenetic mark employed by the cell to control gene expression without altering the genetic sequence [6], although the relationship between the two data types is exceptionally complex [7–10]. Given the flexibility for modification in the epigenome, these methylation events may also serve to provide insight into the environmental exposures sustained by the cell [11], and as potential biomarkers of early cellular transformations [12]. In fact, many examples exist, particularly in the cancer field, for the exploitation of DNA methylation in the diagnosis, prognosis, and prediction of drug response in disease [12, 13], and as possible therapeutic targets [14, 15].

Here, we subject a subset of our highly annotated cohort samples to DNA methylation arrays and investigate differences in the placental methylome between our previously identified transcriptional clusters, as well as relationships between the two data types. Furthermore, by assessing epigenetic changes associated with the observed pathological gene expression, we also attempt to discover novel therapeutic targets for the various PE subtypes.

## Methods

### Sample selection

A total of 48 (out of 157) placentas from our highly annotated cohort purchased from the Research Centre for Women’s and Infants’ Health (RCWIH) BioBank [3] were selected for DNA methylation analysis (19 from transcriptional cluster 1, 19 from transcriptional cluster 2, 5 from transcriptional cluster 3, and 5 from transcriptional cluster 5), using the *sample* function in R 3.1.3 (Additional file 1: Figure S1). The selected number of samples per cluster is approximately representative of the sample distribution in the full placental dataset, with the condition of a minimum of five samples per cluster. Our cohort selection and tissue sampling methods have been previously described [3]. Placentas demonstrating signs of chorioamnionitis or belonging to the chorioamnionitis-associated transcriptional cluster 4 [3] were not included as these are a known entity, independent of preeclampsia (Additional file 1: Figure S1). Clinical differences between these 48 patients only were assessed using Kruskal-Wallis rank sum, Wilcoxon rank sum, and Fisher’s exact tests, as appropriate.

### Methylation arrays and data processing

DNA was isolated from the 48 placentas by ethanol precipitation with the Wizard® Genomic DNA Purification Kit from Promega and quantified by a NanoDrop 1000 spectrophotometer. A total of 750 ng of DNA per sample was bisulfite converted using the EZ Gold DNA methylation kit (Zymo) and assessed for methylation status with Infinium HumanMethylation450 arrays from Illumina. This array covers CpG islands (tight clusters of CpG sites) as well as shores (up to 2 kb from CpG islands), shelves (2–4 kb from CpG islands) and open sea (> 4 kb from CpG islands) [16]. Arrays were scanned by an Illumina HiScan 2000. This methylation data was also used as a validation cohort in [17].

The resulting IDAT files were loaded into R using the *champ.load* function (ChAMP library) [18], excluding low quality probes with a detection *p* value above 0.01 in more than one sample or a beadcount < 3 in at least 5% of samples (*N* = 1940). Probes known to bind sex chromosomes, cross-hybridize to multiple locations, or target a single-nucleotide polymorphism (SNP) were removed, based on previous annotation [19, 20]. This left 410,664 probes for DNA methylation analysis. The samples underwent functional normalization with the *preprocessFunnorm* function [21], which is an extension of quantile normalization utilizing the control probes on the array, applied separately to the methylated and unmethylated intensities, type I and type II signals, and the male and female samples. The data was then batch corrected for slide and array position using the ComBat function (*sva* library) [22] without accounting for any outcome of interest or other covariates to obtain the most unbiased results. All analysis was performed using M values to improve the statistical calculation of differential methylation [23, 24], although beta values are also included in the tables for biological interpretation.

### Gene expression data processing

Our entire 157 placenta dataset was previously hybridized against Human Gene 1.0 ST Array chips from Affymetrix [3]. The resulting microarray CEL files for the 48 placentas assessed for methylation in the current study were loaded into R, and normalized and converted to log2 values using the *affy* library [25]. Expression values annotated to the same gene symbol were merged to a mean value, and genes with expression in the lowest quartile were filtered out to reduce confounding by background noise, using the *varFilter* function.

### Identification of differentially methylated positions

**Fig. 1:**
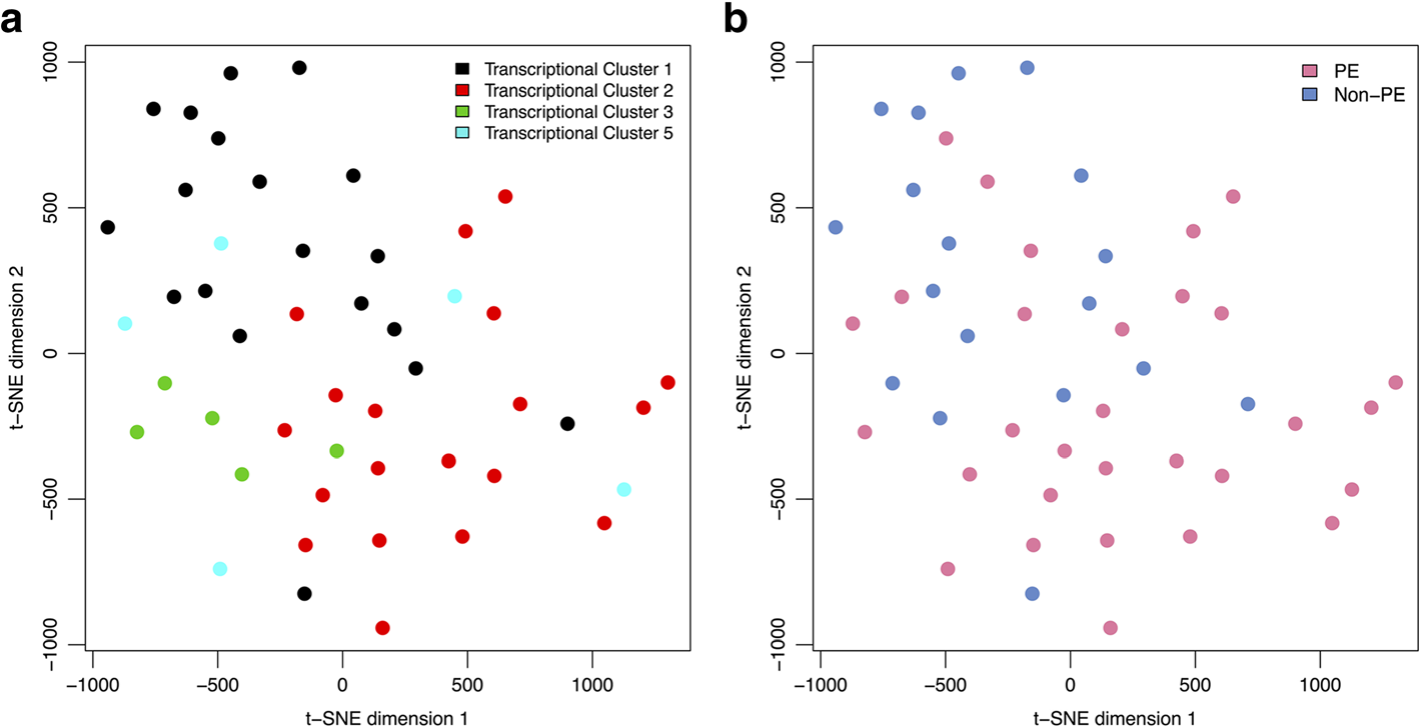
t-Distributed Stochastic Neighbor Embedding (t-SNE) visualization of the methylation data in the 48 placenta samples. **a** Transcriptional clusters 1 (black, *N* = 19), 2 (red, *N* = 19), and 3 (green, *N* = 5) continued to display molecular similarity to each other based on the DNA methylation data alone, indicating that methylation plays a significant role in the development of these three clusters. Cluster 5 samples (cyan, *N* = 5), however, were found dispersed across the methylation plot, no longer forming a united group. **b** In general, preeclamptic (PE) placentas (pink) were found on the bottom half of the t-SNE plot, while the non-PE samples (blue) were predominately observed on the top half. However, the cluster 1 PE patients fully integrated with their co-clustering controls by methylation

The global relationships between the 48 samples based on the DNA methylation information alone were visualized using t-distributed stochastic neighbor embedding (t-SNE; *tsne* library) [26] with a perplexity of 10. Samples belonging to our previously described transcriptional clusters 2, 3, and 5 were compared to cluster 1 placentas (with a “healthy” transcriptional profile) to identify differentially methylated positions, using the *limma* library [27]. The entire cluster 1 was employed as the comparison group after confirming that no significant differentially methylated positions exist between the PE and normotensive controls within cluster 1 by *limma*, and no segregation of these phenotype groups within cluster 1 were observed by t-SNE (Fig. 1). Linear modeling, compared to cluster 1, was performed both with and without controlling for fetal sex (male or female) and/or gestational age (GA) at delivery (26–40 weeks) to investigate the impact of these variables on each cluster. Fetal sex was still considered despite the removal of the sex chromosomes from the analysis due to likely persistent differences on the autosomes [28, 29]. Sites were considered differentially methylated at a false discovery rate (FDR) corrected *q* value < 0.05, and groups of significant positions were noted when at least three significant sites were identified within 1000 base pairs of each other.

### Probe annotation and epigenetic regulation of gene expression

All DNA methylation probes were assigned to enhancer regions, CpG regions (island, shore, shelf, or open sea), and gene-centric locations (TSS1500: 200-1500 nucleotides upstream of the transcriptional start site (TSS); TSS200: TSS to 200 nucleotides upstream of the TSS; 5′ untranslated region (UTR); 1st exon; gene body; 3′UTR; and intergenic region (IGR)) based on the *IlluminaHumanMethylation450kanno.ilmn12.hg19* library. A number of sites (*N* = 45,354) were linked to multiple genes or gene aliases, and all possible associations were maintained. Probes found in the IGR were assigned to the gene with the closest TSS. Trends in significant probe positions were assessed by Fisher’s exact tests.

Sites identified as significantly differentially methylated in transcriptional cluster 2 or 3 placentas, compared to cluster 1 samples, were investigated for linear correlations between the M values and the corresponding log2 gene expression values within the relevant two clusters. Correlations were considered significant at a FDR < 0.05 and correlation groups were compared by Fisher’s exact tests.

### Significance-based modules integrating the transcriptome and epigenome (SMITE)

Differential gene expression between the current subset of transcriptional cluster 2 and 3 samples, compared to cluster 1 placentas, was obtained using the *limma* library [27], controlling for fetal sex and gestational age. Using the hg19 genome build within the *SMITE* library [30] in R 3.3.2, a framework was constructed where each gene was associated with a promoter region (+/− 1500 bp from the TSS) and a gene body region (TSS + 1500 bp to TES). The fetal sex and GA-corrected gene expression and methylation results for clusters 2 and 3 (compared to cluster 1) were then separately integrated into the framework, and the adjusted and combined methylation *p* values in the promoter and body gene regions were obtained using Stouffer’s method, weighted by effect strength. The relationship between expression and methylation was set to “bidirectional” in both gene regions to avoid biasing the results, and genes were scored based on a weighted significance value (0.4 for expression, 0.4 for promoter methylation, and 0.2 for body methylation). Gene scores were considered significant at a nominal *p* value < 0.05. Functional modules of genes in transcriptional clusters 2 and 3 were then identified based on these gene scores, a Reactome protein-protein interaction graph [31], and the spin-glass network algorithm. Significant modules (nominal *p* < 0.05 and 10–500 genes) were subjected to KEGG pathway enrichment analysis within the *SMITE* library [30] and terms with a FDR < 0.05 were held as significant.

## Results

### Clinical characteristics and global methylation patterns

Within this subset of 48 cases, transcriptional cluster 1 patients (*N* = 19) remained the healthiest clinically, with the latest gestational ages at delivery and the highest rates of average-for-gestational-age (AGA) infants (95%) (Additional file 2: Table S1 and Additional file 3: Table S2). Of these cluster 1 patients, 32% (6/19) were associated with a diagnosis of PE, though none had co-occurring fetal growth restriction. Cluster 2 (*N* = 19) and cluster 3 (*N* = 5) samples demonstrated substantially worse clinical outcomes, with abnormal Doppler ultrasound results, early deliveries (mean = 31 weeks), and low placental and newborn weights (mean z-scores < − 1.4) (Additional file 2: Table S1 and Additional file 3: Table S2). In cluster 2, 89% (17/19) were diagnosed with PE and exhibited the highest maternal blood pressures (average maximum systolic pressure = 175 mmHg) and proteinuria levels (average maximum = + 3.5). Cluster 3 (60% PE (3/5)) was more strongly associated with poor fetal growth, with the largest portion of small-for-gestational-age (SGA) infants (80%) and NICU transfers after delivery (80%). Cluster 5 patients (*N* = 5, 80% PE) continued to display no unique clinical association (Additional file 2: Table S1 and Additional file 3: Table S2). These results are consistent with our previous observations in the full transcriptional clusters [3].

When the global relationships between these 48 patients were visualized using t-SNE of the DNA methylation data only, transcriptional cluster 1, 2, and 3 samples continued to demonstrate molecular similarity to each other (Fig. 1a), indicating that methylation plays an important role in the development of these three clusters. Cluster 5 samples, however, were found dispersed across the methylation plot, no longer forming a united group (Fig. 1a).

### Differentially methylated positions between transcriptional clusters

To identify potential epigenetic markers related to our transcriptional clusters, placentas belonging to clusters 2, 3, and 5 were independently assessed for differentially methylated positions (CpG sites) compared to the healthier cluster 1. When fetal sex and gestational age were not considered, this revealed a total of 66,837 positions (53,635 hypo- and 13,202 hyper-) with significantly divergent methylation in transcriptional cluster 2 samples compared to cluster 1 (FDR < 0.05; Additional file 4: Table S3). When fetal sex (*p* = 0.51 between clusters 1 and 2) was integrated into the model, this number was reduced to 64,025, whereas when gestational age (*p* < 0.01 between clusters 1 and 2) alone was incorporated, only 8711 significant positions were observed. However, when these two covariates were simultaneously included in the model, the number of significant sites was 8763 (3310 hypo- and 5453 hyper-) (Table 1 and Additional file 4: Table S3). Similar to the reference distribution across the full set of possible probes, the majority of these (fetal sex and gestational age controlled) significant sites were located in a gene body or an intergenic region (all *p* > 0.05; Additional file 5: Figure S2a). Conversely, substantially fewer significant positions were annotated to a CpG island (12% versus 34%; *p* < 0.01) and considerably more to the CpG open sea (49% versus 33%; *p* = 0.03) than the distribution of the array as a whole (Additional file 5: Figure S2b). Furthermore, 8% (735/8763) of these significant cluster 2 sites were found in a group of at least three significant positions within a span of 1000 base pairs, which were, unsurprisingly, often associated with a CpG island or shore region (*p* < 0.01) (Table 1 and Additional file 4: Table S3).

**Table 1:** Top 20 significantly differentially methylated sites in transcriptional cluster 2 placentas (*N* = 19) compared to transcriptional cluster 1 placentas (*N* = 19), corrected for fetal sex and gestational age at delivery

Probe	Delta M	Average M	Delta β	Average β	FDR *q* value	Gene(s)	Location(s)^a^	Enhancer	Group^b^
cg10900537	0.41	2.75	0.03	0.87	9.93E-04	FOXN3	Body-open sea	True	No
cg18498598	0.35	1.80	0.03	0.78	2.15E-03	CUX1	Body-open sea	False	No
cg11235787	0.36	1.72	0.04	0.77	2.15E-03	MIR195	Body-shelf	False	Yes
cg17850498^c^	− 0.93	1.58	− 0.14	0.74	2.15E-03	ECE1	Body-open sea	True	No
cg01938025	0.62	2.62	0.05	0.86	2.15E-03	SKI	Body-shelf	False	No
cg22807822	0.61	3.49	0.03	0.92	2.15E-03	KANK2	Body-shore	False	Yes
cg14601621	0.54	1.79	0.08	0.77	2.15E-03	C9orf3	3′UTR-island	False	Yes
cg00483891	− 0.55	1.90	− 0.06	0.79	2.15E-03	CCDC115	Body-shore	False	No
cg10994126	− 0.63	− 0.23	− 0.13	0.46	2.92E-03	PAPPA2	1stExon-open sea	False	No
cg17107691	0.76	3.14	0.05	0.89	2.92E-03	KANK2	Body-shore	False	Yes
cg01412654	−0.56	−0.38	−0.11	0.44	2.92E-03	PPARG	TSS1500-shore	False	No
cg05359207	−0.52	−2.62	− 0.05	0.14	2.92E-03	ZNF217	Body-shore	False	No
cg06917772	−0.35	−1.33	− 0.06	0.29	2.92E-03	MIR3167	IGR-shore	True	No
cg24787238	0.34	1.29	0.04	0.71	2.92E-03	MAD1L1	Body-open sea	True	No
cg21564965	0.53	4.73	0.01	0.96	2.92E-03	ARHGAP23	Body-open sea	False	No
cg13562353	−0.53	1.40	−0.09	0.72	2.92E-03	CCL27	TSS200-shelf	False	No
cg26897909	0.45	1.85	0.04	0.78	2.92E-03	SRGAP2	Body-open sea	True	No
cg09106999	−0.44	−2.01	−0.05	0.20	2.92E-03	CDK2 SILV PMEL	TSS1500-shore TSS1500-shore TSS1500-shore	False	No
cg02006426	−0.39	−0.23	−0.05	0.46	2.92E-03	DYSF	IGR-shore	False	No
cg19431235	0.36	1.79	0.04	0.78	2.92E-03	DIAPH3	Body-open sea	True	No

^a^*TSS* transcription start site, *IGR* intergenic region, *UTR* untranslated region

^b^Included in a group of at least three significantly differentially methylated positions within the span of 1000 base pairs

^c^Also significantly differentially methylated in cluster 3 compared to cluster 1

In cluster 3 placentas, 13,348 positions were differentially methylated (9084 hypo- and 4264 hyper-) compared to cluster 1 (FDR < 0.05) without accounting for fetal sex and GA (Additional file 6: Table S4). The inclusion of fetal sex (*p* = 0.12 between clusters 1 and 3) dropped this number to 4343, while accounting for gestational age (*p* = 0.02 between clusters 1 and 3) only in the model reduced the significant positions to 1749. When differences in both these variables were considered, the number of significantly altered sites in transcriptional cluster 3 further decreased to 340 (164 hypo- and 176 hyper-) (Table 2 and Additional file 6: Table S4). The dispersion of these probes was very similar to the results observed in cluster 2: within the gene-based regions, the (fetal sex and GA corrected) significant sites were randomly distributed (all *p* > 0.05; Additional file 7: Figure S3a); however, the number of probes annotated to CpG islands was lower than random (14% versus 34%; *p* < 0.01) and those located in the CpG open sea was higher (51% versus 33%; *p* = 0.01) (Additional file 7: Figure S3b). Additionally, 5% (16/340) of these cluster 3 sites were involved in a group of significant positions that were again more likely to be associated with a CpG island region (*p* < 0.01) (Table 2 and Additional file 6: Table S4).

**Table 2:** Top 20 significantly differentially methylated sites in transcriptional cluster 3 placentas (*N* = 5) compared to transcriptional cluster 1 placentas (*N* = 19), corrected for fetal sex and gestational age at delivery

Probe	Delta M	Average M	Delta β	Average β	FDR *q* value	Gene(s)	Location(s)^a^	Enhancer	Group^b^
cg22131172	0.79	−3.59	0.04	0.08	1.58E-02	C13orf29 LINC00346	TSS1500-open sea TSS1500-open sea	False	Yes
cg24079702	0.50	−5.87	0.01	0.02	1.94E-02	FHL2	TSS200-island	False	No
cg10959820	0.75	−3.98	0.03	0.06	1.94E-02	RGS12	IGR-shelf	True	No
cg10319331	0.63	−3.84	0.02	0.07	1.94E-02	TMEM132B	Body-open sea	False	No
cg05929019	−0.80	−2.79	−0.05	0.13	1.94E-02	LAMC2	TSS200-open sea	False	No
cg22790835	0.60	−2.93	0.04	0.12	1.94E-02	C13orf29 LINC00346	TSS1500-open sea TSS1500-open sea	False	Yes
cg14557185	0.78	−5.55	0.01	0.02	1.94E-02	WWTR1	Body-island	False	Yes
cg00296578	0.74	−3.60	0.04	0.08	1.94E-02	CRIM1	Body-open sea	True	No
cg21834463	−0.47	3.31	−0.03	0.91	1.94E-02	SGK1	Body-open sea	True	No
cg12634306	1.44	−4.70	0.05	0.04	1.94E-02	HEYL	Body-open sea	True	No
cg22342100	0.83	−4.06	0.03	0.06	1.94E-02	KLHL38	TSS1500-open sea	False	No
cg27570256	−0.67	1.50	−0.10	0.74	1.94E-02	LOC100270710	TSS200-shelf	True	No
cg24741430	0.66	−3.09	0.05	0.11	1.94E-02	SMAD6	IGR-open sea	True	No
cg04082512	−0.43	0.77	−0.07	0.63	1.94E-02	GSE1	IGR-open sea	True	No
cg19458020	0.89	−4.52	0.03	0.04	2.14E-02	RARA	TSS1500-island	True	No
cg25892587	0.67	−3.41	0.04	0.09	2.14E-02	KLF6	IGR-open sea	True	No
cg07605236^c^	−1.22	2.60	−0.11	0.85	2.14E-02	SFXN5	TSS1500-shore	False	No
cg19478410	−0.61	2.34	−0.07	0.83	2.16E-02	SYT13	IGR-open sea	False	No
cg13250752	0.57	−1.98	0.06	0.20	2.16E-02	PCDH18	IGR-open sea	True	No
cg20669292	−1.03	3.93	−0.05	0.94	2.16E-02	PLEKHH3	Body-island	True	No

^a^*TSS* transcription start site, *IGR* intergenic region

^b^Included in a group of at least three significantly differentially methylated positions within the span of 1000 base pairs

^c^Also significantly differentially methylated in cluster 2 compared to cluster 1

Compared to transcriptional cluster 1, only four CpG sites were initially identified as differentially methylated in cluster 5 placentas (one hypo- and three hyper-) (FDR < 0.05; Additional file 8: Table S5), and this number became zero when fetal sex and gestational age were included. This indicates that the gene expression changes that define this cluster are not associated with consistent DNA methylation differences. As such, cluster 5 samples were not investigated further for epigenetic regulation.

### Specific functional epigenetic modifications

**Fig. 2:**
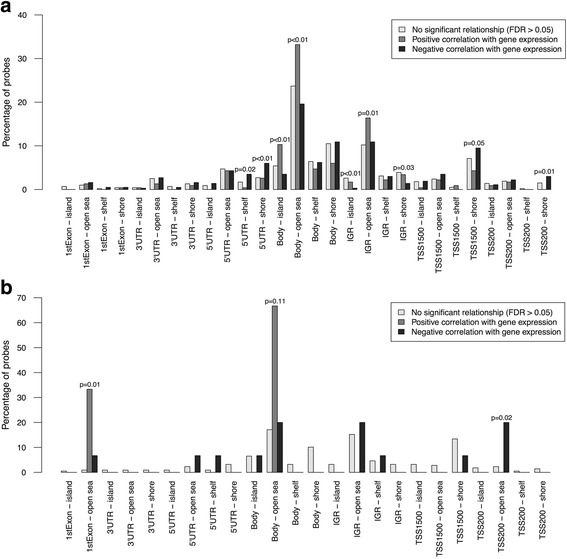
Distribution of the significantly differentially methylated positions in transcriptional cluster 2 and 3 placentas, compared to cluster 1, in terms of their linear relationships to gene expression. **a** Within the 8763 (fetal sex and gestational age corrected) significant sites identified in transcriptional cluster 2, positively correlating positions were more frequently found in a CpG island within a gene body or in the CpG open sea in a gene body or intergenic region (IGR). Significant methylation sites with a negative relationship to gene expression were commonly annotated to a CpG shelf region in a 5′ untranslated region (UTR) or a CpG shore region in a 5′UTR, transcription start site (TSS)1500, or TSS200. **b** Within the 340 cluster 3 (fetal sex and gestational age corrected) significant sites, only three demonstrated a strong positive relationship with expression: one was in the CpG open sea of AFF3′s 1st exon and the other two were in the CpG open sea of the MGST1 gene body. Negatively correlating positions were more frequently annotated to an open sea region in a TSS200, although several were also in gene bodies or the IGR. Non-significant correlations are shown in light gray, positive correlations are in medium gray, and negative correlations are in dark gray. Nominal *p* values were obtained from Fisher’s exact tests. *P* values > 0.05 are not shown

In order to identify individual epigenetic changes involved in the transcriptional formation of clusters 2 and 3, significantly differentially methylated sites in these samples compared to cluster 1 were assessed for correlating changes in placental gene expression. Of the 66,837 identified significant positions in transcriptional cluster 2 (before correction for fetal sex and GA), correlative analysis with the expression of all available associated genes revealed only 5% with a strong linear relationship (FDR < 0.05; Additional file 9: Table S6). When restricted to the 8763 sites that maintained a significant difference between clusters 1 and 2 after correction for both fetal sex and GA, 9% of potential DNA methylation values exhibited a significant linear relationship with gene expression (FDR < 0.05; Table 3 and Additional file 9: Table S6). Positively correlating positions were more frequently found in a CpG island within a gene body (*p* < 0.01) or in the CpG open sea in a gene body (*p* < 0.01) or intergenic region (*p* = 0.01) (Fig. 2a). Sites with a negative relationship to gene expression were commonly annotated to a CpG shelf region in a 5′UTR (*p* = 0.02) or a CpG shore region in a 5′UTR (*p* < 0.01), TSS1500 (*p* = 0.05), or TSS200 (*p* = 0.01) (Fig. 2a). Most significantly correlating positions within the CpG open sea of a gene body or intergenic region were also associated with an enhancer region (72%; *p* < 0.01 compared to the other CpG/gene regions).

**Table 3:** Top 20 significant gene expression correlations associated with the 8763 significantly differentially methylated sites in transcriptional cluster 2 placentas (*N* = 19) compared to transcriptional cluster 1 placentas (*N* = 19), corrected for fetal sex and gestational age at delivery

Probe	Gene	Location^a^	Enhancer	Pearson r	FDR *q* value
cg23677911	GALNT2	Body-open sea	True	− 0.81	3.53E-06
cg26333638	HEXB	Body-shore	False	− 0.81	3.53E-06
cg04858987	SH3BP5	Body-open sea	True	− 0.78	1.34E-05
cg13553455	COL17A1	TSS1500-open sea	False	− 0.78	1.34E-05
cg16557964	TMEM45A	5′UTR-open sea	True	− 0.77	1.68E-05
cg19140548	SH3PXD2A	Body-open sea	True	− 0.77	2.50E-05
cg15700009	LDHA	TSS1500-shore	False	− 0.76	2.70E-05
cg23730027	FLNB	Body-island	False	− 0.76	2.93E-05
cg18444702	SH3BP5	Body-open sea	True	− 0.75	4.27E-05
cg17338821	FLNB	Body-shore	True	− 0.75	5.14E-05
cg25549791	GALE	TSS200-shore	False	− 0.74	5.49E-05
cg14019050	ABCA1	TSS1500-island	False	− 0.74	5.49E-05
cg19512693	FLT1	Body-open sea	True	0.74	6.15E-05
cg04704064	SCARB1	IGR-island	False	0.74	6.29E-05
cg00411097	TMEM184A	1stExon-open sea	True	− 0.73	7.24E-05
cg11079619	INHBA	5′UTR-shelf	False	− 0.73	7.32E-05
cg00513984	SCARB1	IGR-island	False	0.73	7.32E-05
cg26509870	PHYHIP	IGR-shelf	False	− 0.73	8.19E-05
cg06531595	PDE5A	Body-open sea	True	− 0.72	9.89E-05
cg18874575	ZNF559	3′UTR-open sea	False	0.72	9.89E-05

^a^*TSS* transcription start site, *IGR* intergenic region, *UTR* untranslated region

In transcriptional cluster 3, the 13,348 significant sites compared to cluster 1 (before correction for fetal sex and GA) showed a strong linear relationship to gene expression only 2% of the time (Additional file 10: Table S7). This value increased to 8% when the analysis was restricted to the 340 positions that were significantly differentially methylated between clusters 1 and 3 when simultaneously controlling for fetal sex and GA (Table 4). Only three sites demonstrated a strong positive relationship with expression: one was in the CpG open sea of *AFF3*’s 1st exon (*p* = 0.01), which was not annotated as an enhancer region, and the other two were in the CpG open sea of the *MGST1* gene body (*p* = 0.11) in an enhancer (Fig. 2b). Negatively correlating positions were more frequently associated with an open sea region in a TSS200 (*p* = 0.02), although several were also in gene bodies or the IGR (Fig. 2b).

**Table 4:** All significant gene expression correlations associated with the 340 significantly differentially methylated sites in transcriptional cluster 3 placentas (*N* = 5) compared to transcriptional cluster 1 placentas (*N* = 19), corrected for fetal sex and gestational age at delivery

Probe	Gene	Location^a^	Enhancer	Pearson r	FDR *q* value
cg03983223	WIPF1	1stExon-open sea	FALSE	− 0.74	4.45E-03
cg05544807	DNMT3A	Body-island	FALSE	− 0.73	4.45E-03
cg22462240	LGALS3BP	IGR-open sea	FALSE	− 0.74	4.45E-03
cg18275589	DAB2	IGR-shelf	FALSE	− 0.72	5.02E-03
cg09258479	PDZK1IP1	TSS200-open sea	FALSE	− 0.69	7.40E-03
cg07593977	CTSB	IGR-open sea	TRUE	− 0.69	7.40E-03
cg24506086	TEAD1	Body-open sea	TRUE	− 0.66	1.40E-02
cg17850498	ECE1	Body-open sea	TRUE	− 0.65	1.65E-02
cg07349094	AFF3	1stExon-open sea	FALSE	0.64	1.65E-02
cg03821121	MICAL2	5′UTR-open sea	TRUE	− 0.65	1.65E-02
cg04885072	MGST1	Body-open sea	TRUE	0.63	1.94E-02
cg00874480	MGST1	Body-open sea	TRUE	0.62	2.45E-02
cg11535839	FOSL2	IGR-open sea	FALSE	− 0.61	2.59E-02
cg05305434	LSP1	TSS200-open sea	FALSE	− 0.61	2.72E-02
cg23170988	SNCG	Body-open sea	FALSE	− 0.60	3.17E-02
cg05929019	LAMC2	TSS200-open sea	FALSE	− 0.59	3.75E-02
cg15300730	ZFP36L2	TSS1500-shore	FALSE	− 0.58	3.75E-02
cg22234930	PKM	5′UTR-shelf	FALSE	− 0.58	3.83E-02

^a^*TSS* transcription start site, *IGR* intergenic region, *UTR* untranslated region

### Integrated functional gene modules

**Fig. 3:**
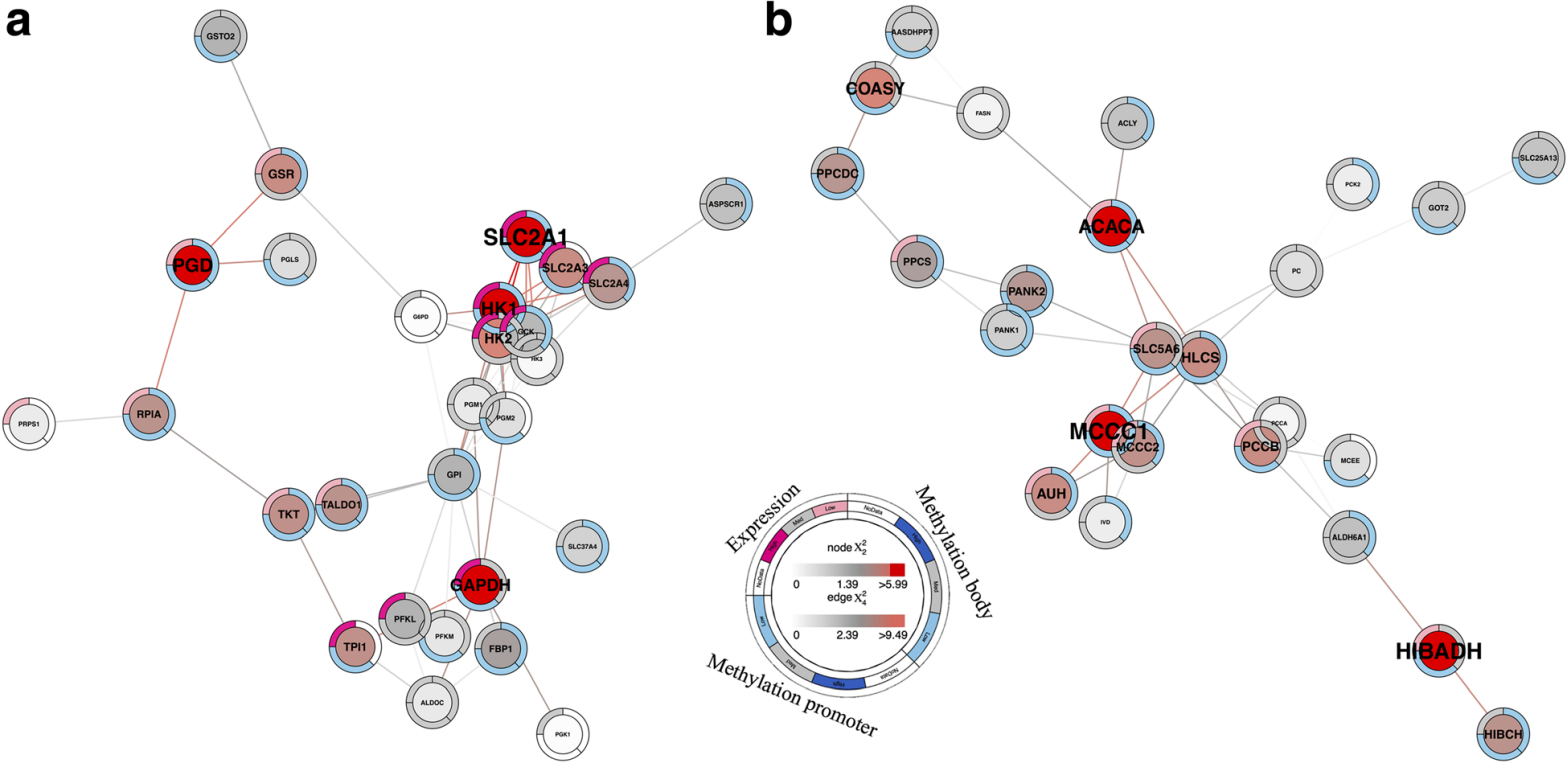
Example SMITE modules. **a** Cluster 2 module 6 (*N* = 28 genes; *p* = 0.02) built around *SLC2A1* and *HK1* genes and involved in carbohydrate metabolism. **b** Cluster 3 module 11 (*N* = 25 genes; *p* = 0.04) built around *MCCC1* and *ACACA* genes and involved in amino acid and biotin metabolism. Expression is displayed on the top left edge of each gene circle (upregulated: dark pink; downregulated: light pink; gray: not significant; white: no data), and combined promoter and body methylation are displayed on the bottom left and top right of each circle, respectively (hypermethylated: dark blue; hypomethylated: light blue; gray: not significant; white: no data), compared to cluster 1. The symbol text sizes and center node colors are based on the total gene score (low (gray) to high (red)) and the edge colors are representative of the strength of the associations between the genes (low (gray) to high (red)). The remaining modules are shown in Additional file 11: Figure S4 and Additional file 14: Figure S5

Lastly, in order to reveal significant functional modules of genes within clusters 2 and 3, their differential gene expression and differential gene promoter and body methylation information, compared to cluster 1 and corrected for fetal sex and GA, were subjected to Significance-based Modules Integrating the Transcriptome and Epigenome (SMITE) analysis [30]. Transcriptional cluster 2 contained 9 significant integrated gene modules (*p* < 0.05), consisting of 18–149 genes each (Fig. 3a and Additional file 11: Figure S4). Modules in this cluster with unique genes (1, 4, and 6) were associated with TGF-beta signaling, cell adhesion, endocytosis, leukocyte transendothelial migration, and carbohydrate metabolism (Additional file 12: Table S8). Module 3 genes were contained within module 2, and these were involved in focal adhesion and regulation of the actin cytoskeleton. Modules 5 and 9 were associated with lipid metabolism, while modules 7 and 8 were linked to oxidative phosphorylation and the citrate cycle. The significantly deregulated genes in cluster 2, based on the integrated epigenetic and transcriptional scores, and their module inclusions, are shown in Additional file 13: Table S9.

In contrast, cluster 3 consisted of 11 significant gene modules (*p* < 0.05), made up of 24–293 genes each (Fig. 3b and Additional file 14: Figure S5). Modules 1, 4, 5, 6, and 7 displayed varying degrees of gene overlap and were all involved in TGF-beta signaling, focal adhesion, and glycosaminoglycan biosynthesis (Additional file 12: Table S8). Modules 2 and 3 were linked to antigen processing/presentation and allograft rejection, while modules 8 and 9, with ~ 74% gene overlap, were associated with cytokine-cytokine receptor interaction and Jak-STAT signaling. Modules in this cluster with unique genes (10 and 11) were involved in purine, amino acid, and biotin metabolism. The significantly deregulated genes in transcriptional cluster 3, based on both gene expression and methylation, and their module inclusions, are also shown in Additional file 13: Table S9.

## Discussion

Our previous work unbiasedly investigating the placental heterogeneity observed in preeclampsia [3] revealed five transcriptional clusters, including four subtypes of PE placentas. However, while gene expression microarrays are an invaluable tool for understanding disease, it is also possible that, in some cases, an alternate level of molecular information is highly involved in the development of the pathology. Combined epigenetic and expression analysis of the same preeclamptic placentas has only ever been performed for a small number of samples [32, 33] or genes [34]. We, therefore, predicted that the integration of matched genome-wide DNA methylation data would further improve our understanding of these placentas, and allow us to investigate both the mechanisms underlying the formation of the transcriptional clusters and the associations between the multi-molecular data.

Overall, we found that the relationships between the transcriptional cluster 1–3 samples were still visible within the DNA methylation information, indicating a significant global relationship between the two data types in these samples. Cluster 5 placentas, on the other hand, no longer formed a distinct group by methylation. This is unsurprising given that this data type is known to be fairly robust to copy number abnormalities [35], the driving force behind the molecular formation of this cluster.

Within transcriptional clusters 2 and 3, controlling for fetal sex and gestational age in the identification of differentially methylated sites, compared to the healthier cluster 1, dramatically reduced the number of significant sites (66,837 to 8763 in cluster 2; 13,348 to 340 in cluster 3). However, it predominately corrected the observed imbalance in the direction of change (80% hypomethylated to 38% hypomethylated in cluster 2; 68% hypomethylated to 48% hypomethylated in cluster 3). Since both clusters 2 and 3 are significantly younger than cluster 1 (*p* < 0.01 and *p* = 0.02, respectively), this fits with the knowledge that placentas become progressively more methylated with time [36], while in cluster 3, a moderate bias in fetal sex (*p* = 0.12) may have also been involved. Additionally, controlling for fetal sex and GA substantially increased the proportion of significant sites that showed a strong linear relationship with gene expression (5% to 9% in cluster 2; 2% to 8% in cluster 3), thereby confirming that a large number of sites in the genome undergo DNA methylation changes in response to differences in these two factors that are independent of epigenetic regulation and gene expression [9, 36, 37].

An additional result of interest was the CpG distribution of significant positions found in transcriptional clusters 2 and 3. CpG islands are most commonly associated with the regulation of gene expression, especially when located in the gene’s promoter region [8, 38]. We discovered that substantially fewer of the significant sites were mapped into CpG islands than anticipated, based on the reference distribution of all potential CpG sites, although those that were annotated to islands were, unsurprisingly, often found in close proximity to each other. Instead, the majority of significant positions were associated with CpG open sea enhancer regions. This is consistent with a previous report of enrichment of altered DNA methylation at enhancers and low CpG density regions in early-onset preeclamptic placentas [33]. These open sea enhancer regions, when significantly associated with gene expression, were generally located in the gene body and exhibited a positive relationship. Sites with a strong negative correlation, on the other hand, were frequently located in the promoter region (TSS200, TSS1500, 5′UTR), as expected, but were annotated to CpG shore regions, not islands. Relationships between CpG shores and gene expression are thought to be in response to the binding of transcription factors and changes in the chromatin structure around the promoter [39, 40].

While the observed proportions of differentially methylated sites that were associated with corresponding changes in gene expression (2–9%) are in line with prior studies [9, 32, 41, 42], this indicates that a large number of significant sites in clusters 2 and 3, compared to cluster 1, show no meaningful relationship to gene expression. Some of these DNA methylation alterations could be the consequence of changes in gene expression or function [29, 43, 44], or an adaptive response to maintain stable or rebalanced expression. They could further be remnants of an earlier developmental process, or the result of environmental exposures or treatments, where the transcriptional evidence is no longer measurable [36]. Furthermore, methylation is involved in a range of functions outside of direct transcriptional regulation, such as genome stability [45], splicing [8, 46], and cellular development [47], while gene expression can be regulated by a number of other factors, such as microRNAs [48, 49], transcription factors [43, 50], and/or histone modifications [51, 52]. Therefore, it is expected that these two data types would not fully agree at the individual gene level, although altered methylation sites not associated with changes in gene expression could still provide important information about the overall status and gestational history of these pathological placentas.

When the transcriptome and epigenome data was utilized simultaneously in an integrated analysis, this revealed modifications in TGF-beta signaling, cell adhesion and migration, oxidative phosphorylation, and carbohydrate and lipid metabolism pathways in cluster 2 placentas, confirming that a significant global relationship exists between the two data types. Placental dysfunction encompassing dysregulation of these pathways has been extensively described in the classical paradigm of PE pathophysiology and fits with our characterization of cluster 2 patients as demonstrating a “canonical” early-onset form of PE [3, 53–59]. Additionally, a number of the top significant methylation and gene expression correlations in this cluster (cg23730027 and *FLNB*, cg13553455 and *COL17A1*, cg11079619 and *INHBA*, cg19140548 and *SH3PXD2A*, and cg26509870 and *PHYHIP*) have been previously described in a smaller sample set of early-onset PE placentas [33], thus validating these relationships. We also identified several methylation probes in the gene body of *FLT1*, one of the most frequently investigated markers of PE, with a strong positive correlation to expression, as well as one associated site in the IGR with a strong negative correlation. These methylation differences could be involved in or attempting to compensate for the pathologically elevated expression of this gene [2, 3], and are significant findings missed by prior studies that have focused only on *FLT1* promoter methylation in early-onset PE [34].

In cluster 3 samples, integrated alterations were identified involving antigen presentation, allograft rejection, cytokine-cytokine receptor interaction, Jak-STAT and TGF-beta signaling, glycosaminoglycan biosynthesis, and metabolism. These are also in line with our prior transcriptional results in this “immunological” PE group [3], in which we characterized this cluster of patients as demonstrating evidence of maternal anti-fetal/placental rejection. While not as widely discussed in the literature, the primary involvement of heightened immune activation has been described in several previous studies of PE pathophysiology, along with these other metabolic pathways [60–66]. Interestingly, one of the most significant methylation and expression relationships observed in this cluster involved *DNMT3A* (one of the DNA methyltransferase enzymes responsible for de novo methylation): a CpG island site (cg05544807) was hypermethylated in the *DNMT3A* gene body, compared to cluster 1, and demonstrated a negative relationship to expression. While this likely has global implications for the DNA methylation pattern observed in these cluster 3 placentas, decreased expression of *DNMT3A* has been specifically implicated in immunological-associated disorders [67, 68] and abnormal placentation in preeclampsia [69]. Therefore, this CpG site may serve as a potential target for the epigenetic modulation of pathological gene expression in this PE subtype.

Our study also has some inherent limitations. In our previous gene expression analysis, we utilized a large cohort of over 300 placentas to identify clusters and dysregulated pathways between them. Despite our current study being the largest to integrate methylation and transcriptional information in PE, this analysis involved only 48 placentas. Therefore, it is likely to still be underpowered, thus restricting our discovery of epigenetic changes in these samples to those with large effect sizes. As such, a future direction will be the validation of these findings, and perhaps the identification of new significant sites, in a larger cohort of samples. Additionally, as with all investigations of delivered placentas, it is impossible to determine whether the observed molecular modifications are part of the cause or the consequence of the disease process. Finally, our analysis is based on the assumption that the cell composition is the same across all samples. This is probably not the case, as differences in cell ratios can occur for a range of reasons [4, 42, 70–72], including placental maturation or sampling variability. Therefore, some of the epigenetic changes that we are interpreting as being reflective of gene regulation in all cells may instead be due to shifts in cell numbers [29]. However, unfortunately, until individual methylation patterns for all possible placental cell types have been established, this limitation cannot be resolved. This investigation is currently ongoing in our groups.

## Conclusions

Overall, we have improved our understanding of the portion of the divergent gene expression involved in the development of transcriptional clusters 2 and 3 that is associated with changes in DNA methylation, as well as confirmed the lack of true biological cohesion in cluster 5 placentas. Differentially methylated sites in clusters 2 and 3, compared to the healthier cluster 1, may have potential as biomarkers of these patient groups for early screening in maternal serum, whereas specific genes and sets of genes exhibiting a strong epigenetic and transcriptional relationship (either linear or integrated) may serve as therapeutic targets to modify or prevent pathological changes in PE placental groups. However, a further increase in sample size and an assessment of additional modes of gene regulation will be required to fully comprehend the mechanisms underlying these subtypes.

## Additional files

Additional file 1: **Figure S1.** Selected samples for methylation arrays. (PDF 199 kb)

Additional file 2: **Table S1.** Continuous clinical characteristics of the 48 samples across the transcriptional clusters (PDF 72 kb)

Additional file 3: **Table S2.** Categorical clinical characteristics of the 48 samples across the transcriptional clusters (PDF 79 kb)

Additional file 4: **Table S3.** Significantly differentially methylated sites in transcriptional cluster 2 placentas versus transcriptional cluster 1 placentas. (XLSX 9040 kb)

Additional file 5: **Figure S2.** Distribution of significantly differentially methylated positions in transcriptional cluster 2 (versus transcriptional cluster 1) compared to the full set of possible methylation probes. (PDF 559 kb)

Additional file 6: **Table S4.** Significantly differentially methylated sites in transcriptional cluster 3 placentas versus transcriptional cluster 1 placentas. (XLSX 1669 kb)

Additional file 7: **Figure S3.** Distribution of significantly differentially methylated positions in transcriptional cluster 3 (versus transcriptional cluster 1) compared to the full set of possible methylation probes. (PDF 560 kb)

Additional file 8: **Table S5.** Significantly differentially methylated sites in transcriptional cluster 5 placentas versus transcriptional cluster 1 placentas. (XLSX 38 kb)

Additional file 9: **Table S6.** Significant gene expression correlations associated with the significantly differentially methylated sites in transcriptional cluster 2 placentas versus transcriptional cluster 1 placentas. (XLSX 259 kb)

Additional file 10: **Table S7.** Significant gene expression correlations associated with the significantly differentially methylated sites in transcriptional cluster 3 placentas versus transcriptional cluster 1 placentas. (XLSX 63 kb)

Additional file 11: **Figure S4.** Remaining functional SMITE modules identified in cluster 2. (PDF 2447 kb)

Additional file 12: **Table S8.** Significant KEGG pathways associated with the significant SMITE modules in transcriptional clusters 2 and 3 (XLSX 58 kb)

Additional file 13: **Table S9.** Genes with significant integrated gene expression and methylation scores by SMITE analysis in transcriptional clusters 2 and 3. (XLSX 86 kb)

Additional file 14: **Figure S5.** Remaining functional SMITE modules identified in cluster 3. (PDF 4125 kb)

## Abbreviations

aCGH: Array-based comparative genomic hybridization
AGA: Average-for-gestational-age
FDR: False discovery rate
GA: Gestational age
IGR: Intergenic region
PE: Preeclampsia
RCWIH: Research Centre for Women’s and Infants’ Health
SGA: Small-for-gestational-age
SMITE: Significance-based Modules Integrating the Transcriptome and Epigenome
SNP: Single-nucleotide polymorphism
TES: Transcriptional end site
t-SNE: t-distributed stochastic neighbor embedding
TSS: Transcriptional start site
UTR: Untranslated region
