# Supplementary material for: Epigenetic regulation of placental gene expression in transcriptional subtypes of preeclampsia
Source: Clin Epigenetics. 2018 Mar 2;10:28. doi: 10.1186/s13148-018-0463-6 (PMC5833042; doi:10.1186/s13148-018-0463-6)
Supplement: Supplementary file 1 — Figure S1. Selected samples for methylation arrays. (PDF 199 kb) [file 13148_2018_463_MOESM1_ESM.pdf]

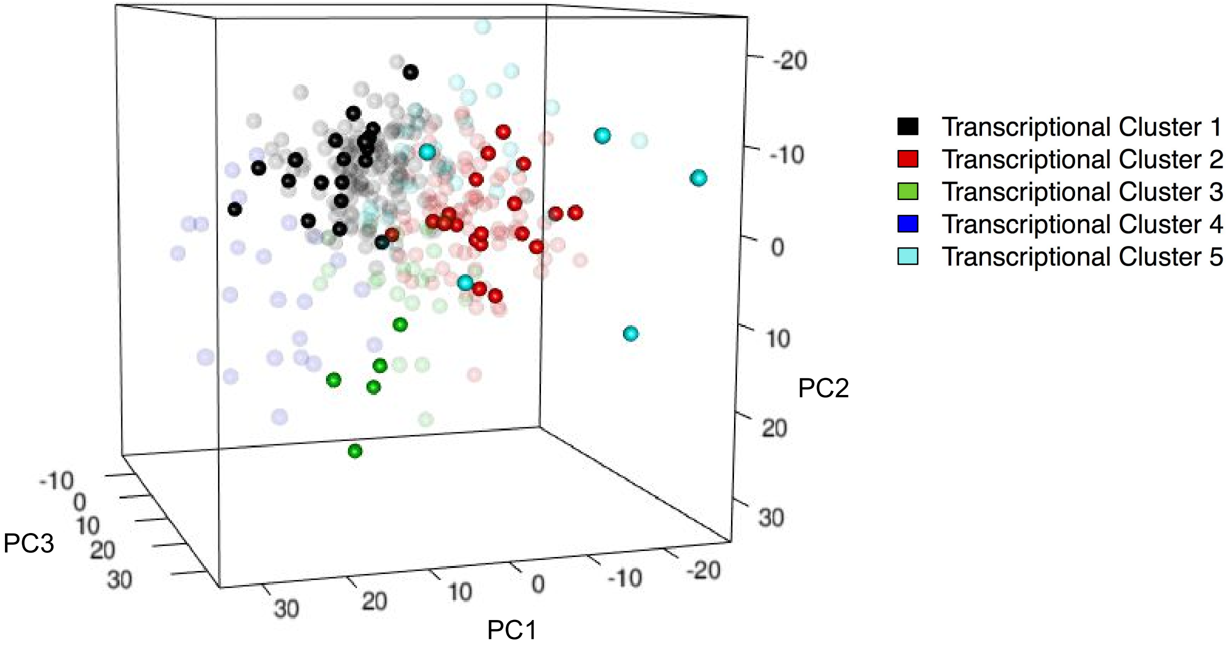

Supplementary Figure 1. Selected samples for methylation arrays. A total of 48 (out of 157) samples from our highly annotated placental data set were selected for DNA methylation analysis (19 from transcriptional cluster 1 (black), 19 from cluster 2 (red), five from cluster 3 (green), and five from cluster 5 (cyan)). Placentas demonstrating signs of chorioamnionitis or belonging to the chorioamnionitis-associated transcriptional cluster 4 (blue) were not included as these are a known entity, independent of preeclampsia. Transparent spheres are unselected samples (109/157) and samples that were used for transcriptional clustering but are not accessible for additional experiments (previously published microarray data) (N=173).
