# Supplementary material for: Epigenetic regulation of placental gene expression in transcriptional subtypes of preeclampsia
Source: Clin Epigenetics. 2018 Mar 2;10:28. doi: 10.1186/s13148-018-0463-6 (PMC5833042; doi:10.1186/s13148-018-0463-6)
Supplement: Supplementary file 2 — Table S1. Continuous clinical characteristics of the 48 samples across the transcriptional clusters (PDF 72 kb) [file 13148_2018_463_MOESM2_ESM.pdf]

Supplementary Table 1. Continuous clinical characteristics of the 48 samples across the transcriptional clusters.

Clinical Attribute	Cluster 1 N=19	Cluster 2 N=19	Cluster 3 N=5	Cluster 5 N=5	P-value
	Mean (SD)[1]				
**Parental demographics**					
Maternal age (years)	32.5 (4.6)	35.1 (5.0)	37.0 (4.3)	31.6 (7.7)	0.19
Maternal BMI (kg/m^2)	26.5 (5.6)	26.1 (3.8)	24.9 (0.5)	24.7 (4.6)	0.79
Maternal height (cm)	163 (6)	163 (7)	161 (5)	161 (11)	0.95
**Uteroplacental blood flow/Ultrasound data**					
Mean uterine artery PI[2]	0.86 (0.25)	1.84 (0.33)	1.64 (0.65)	--	0.03
Max uterine artery PI[2]	1.02 (0.34)	2.20 (0.26)	2.15 (0.79)	--	0.02
Mean umbilical artery PI[2]	0.98 (0.10)	1.56 (0.42)	1.42 (0.35)	1.45 (0.11)	< 0.01
Max umbilical artery PI[2]	1.10 (0.17)	1.71 (0.40)	1.64 (0.32)	1.63 (0.24)	< 0.01
Mean middle cerebral artery PI[2]	1.73 (0.11)	1.47 (0.24)	1.49 (0.15)	1.85 (0.40)	0.03
Min middle cerebral artery PI[2]	1.58 (0.19)	1.26 (0.19)	1.27 (0.13)	1.55 (0.31)	0.02
Mean middle cerebral artery peak systolic velocity (cm/sec)	54.3 (15.1)	51.4 (11.1)	48.2 (12.0)	48.8 (6.3)	0.80
Max middle cerebral artery peak systolic velocity (cm/sec)	58.8 (20.2)	58.8 (11.3)	57.5 (9.6)	54.4 (8.1)	0.94
Mean biophysical profile score (/10)	7.86 (0.34)	7.56 (0.69)	7.23 (0.92)	8.00 (0)	0.21
Min biophysical profile score (/10)	7.56 (0.88)	7.13 (1.02)	6.80 (1.10)	8.00 (0)	0.27
**Preeclampsia diagnostic criteria**					
Max systolic pressure (mm Hg)	137 (24)	175 (17)	160 (22)	154 (23)	< 0.01
Max diastolic pressure (mm Hg)	86 (14)	111 (13)	98 (7)	98 (12)	< 0.01
Max mean arterial pressure (mm Hg)	102 (16)	131 (14)	116 (11)	116 (16)	< 0.01
Max proteinuria level (dipstick)	+1.3 (1.3)	+3.5 (0.9)	+2.0 (1.0)	+2.8 (1.9)	< 0.01
**Fetal demographics**					
Gestational age at delivery (weeks)	36 (3)	31 (3)	32 (4)	33 (3)	< 0.01
Newborn weight z-score	0.15 (1.06)	-1.51 (0.59)	-1.77 (0.46)	-1.25 (1.05)	< 0.01
Apgar score at 1 minute (/10)	8.0 (1.5)	7.5 (1.7)	7.5 (1.7)	7.2 (2.4)	0.50
Apgar score at 5 minutes (/10)	8.8 (0.5)	8.9 (0.4)	8.8 (0.5)	8.4 (1.3)	0.87
**Placental and umbilical cord data**					
Placental weight z-score	-0.06 (0.89)	-1.40 (0.74)	-1.58 (0.70)	-1.00 (0.80)	< 0.01
Placental thickness (cm)	3.18 (0.72)	2.38 (1.43)	1.86 (0.80)	2.20 (0.23)	< 0.01
Placental asymmetry (ratio)	0.12 (0.09)	0.16 (0.09)	0.13 (0.08)	0.21 (0.13)	0.35
Cord insertion distance from placental margin (cm)	3.93 (1.17)	2.32 (0.86)	2.70 (1.03)	2.80 (0.75)	0.01
Cord diameter (cm)	1.30 (0.25)	1.16 (0.42)	0.86 (0.23)	1.16 (0.23)	0.03

[1]Only included if values were available for at least 3 samples in the cluster

[2]PI = pulsatility index
