# Supplementary material for: Epigenetic regulation of placental gene expression in transcriptional subtypes of preeclampsia
Source: Clin Epigenetics. 2018 Mar 2;10:28. doi: 10.1186/s13148-018-0463-6 (PMC5833042; doi:10.1186/s13148-018-0463-6)
Supplement: Supplementary file 3 — Table S2. Categorical clinical characteristics of the 48 samples across the transcriptional clusters (PDF 79 kb) [file 13148_2018_463_MOESM3_ESM.pdf]

Supplementary Table 2. Categorical clinical characteristics of the 48 samples across the transcriptional clusters.

	Cluster 1 N=19	Cluster 2 N=19	Cluster 3 N=5	Cluster 5 N=5	
**Clinical Attribute**	**Percentage of Cluster (n/N)[1]**				**P-value**
**Parental demographics**					
Nulliparous	42 (8/19)	68 (13/19)	20 (1/5)	40 (2/5)	0.19
Previous miscarriage	26 (5/19)	32 (6/19)	40 (2/5)	20 (1/5)	0.96
Previous termination	32 (6/19)	21 (4/19)	20 (1/5)	20 (1/5)	0.95
Previous hypertensive pregnancy	36 (4/11)	100 (4/4)	50 (2/4)	50 (1/2)	0.20
Maternal race					0.08
White	67 (12/18)	37 (7/19)	20 (1/5)	100 (5/5)	--
Black	0 (0/18)	26 (5/19)	40 (2/5)	0 (0/5)	
Asian	28 (5/18)	26 (5/19)	40 (2/5)	0 (0/5)	
East Indian	6 (1/18)	5 (1/19)	0 (0/5)	0 (0/5)	
Paternal race					0.22
White	100 (7/7)	33 (3/9)	33 (1/3)	100 (3/3)	--
Black	0 (0/7)	22 (2/9)	33 (1/3)	0 (0/3)	
Asian	0 (0/7)	22 (2/9)	33 (1/3)	0 (0/3)	
East Indian	0 (0/7)	11 (1/9)	0 (0/3)	0 (0/3)	
Maternal blood type					0.09
A	21 (4/19)	39 (7/18)	60 (3/5)	0 (0/5)	--
B	26 (5/19)	33 (6/18)	40 (2/5)	0 (0/5)	
O	47 (9/19)	22 (4/18)	0 (0/5)	80 (4/5)	
AB	5 (1/19)	6 (1/18)	0 (0/5)	20 (1/5)	
Rh positive	16 (3/19)	6 (1/17)	20 (1/5)	20 (1/5)	0.55
BMI > 25 kg/m^2	58 (11/19)	67 (10/15)	67 (2/3)	60 (3/5)	0.95
Chronic hypertension	11 (2/19)	32 (6/19)	60 (3/5)	20 (1/5)	0.09
**Ultrasound data**					
Placenta position on ultrasound					0.34
Anterior	29 (2/7)	60 (6/10)	67 (2/3)	67 (2/3)	--
Posterior	71 (5/7)	10 (1/10)	33 (1/3)	33 (1/3)	
Amniotic fluid deficiency	0 (0/4)	43 (3/7)	67 (2/3)	0 (0/2)	0.24
**Diagnoses**					
Preeclampsia	32 (6/19)	89 (17/19)	60 (3/5)	80 (4/5)	< 0.01
HELLP[2]	0 (0/19)	26 (5/19)	0 (0/5)	0 (0/5)	0.07
Fetal growth restriction	0 (0/19)	53 (10/19)	80 (4/5)	40 (2/5)	< 0.01
Chorioamnionitis	0 (0/19)	0 (0/19)	0 (0/5)	0 (0/5)	1
**Labor and Delivery**					
Attempted vaginal delivery	63 (12/19)	26 (5/19)	0 (0/5)	60 (3/5)	0.02
Vaginal delivery	42 (8/19)	11 (2/19)	0 (0/5)	20 (1/5)	0.07
Delivery < 34 weeks	21 (4/19)	84 (16/19)	40 (2/5)	60 (3/5)	< 0.01
Delivery < 37 weeks	32 (6/19)	95 (18/19)	100 (5/5)	100 (5/5)	< 0.01

**Fetal demographics**					
Male fetus	68 (13/19)	53 (10/19)	20 (1/5)	40 (2/5)	0.25
AGA[2] (10-90th percentile)	95 (18/19)	32 (6/19)	20 (1/5)	40 (2/5)	< 0.01
SGA[2] (<10th percentile)	0 (0/19)	68 (13/19)	80 (4/5)	60 (3/5)	< 0.01
NICU[2] transfer	5 (1/19)	58 (11/19)	80 (4/5)	20 (1/5)	< 0.01

[1]All available data was utilized, however, information was missing for some samples for some characteristics. Complete data is indicated by N=19 for cluster 1, N=19 for cluster 2, N=5 for cluster 3, and N=5 for cluster 5

[2]HELLP = hemolysis, elevated liver enzymes, and low platelets syndrome; AGA = average-for-gestational-age; SGA = small-for-gestational-age; NICU = neonatal intensive care unit
