# Supplementary material for: Epigenetic regulation of placental gene expression in transcriptional subtypes of preeclampsia
Source: Clin Epigenetics. 2018 Mar 2;10:28. doi: 10.1186/s13148-018-0463-6 (PMC5833042; doi:10.1186/s13148-018-0463-6)
Supplement: Supplementary file 7 — Figure S3. Distribution of significantly differentially methylated positions in transcriptional cluster 3 (versus transcriptional cluster 1) compared to the full set of possible methylation probes. (PDF 560 kb) [file 13148_2018_463_MOESM7_ESM.pdf]

A)

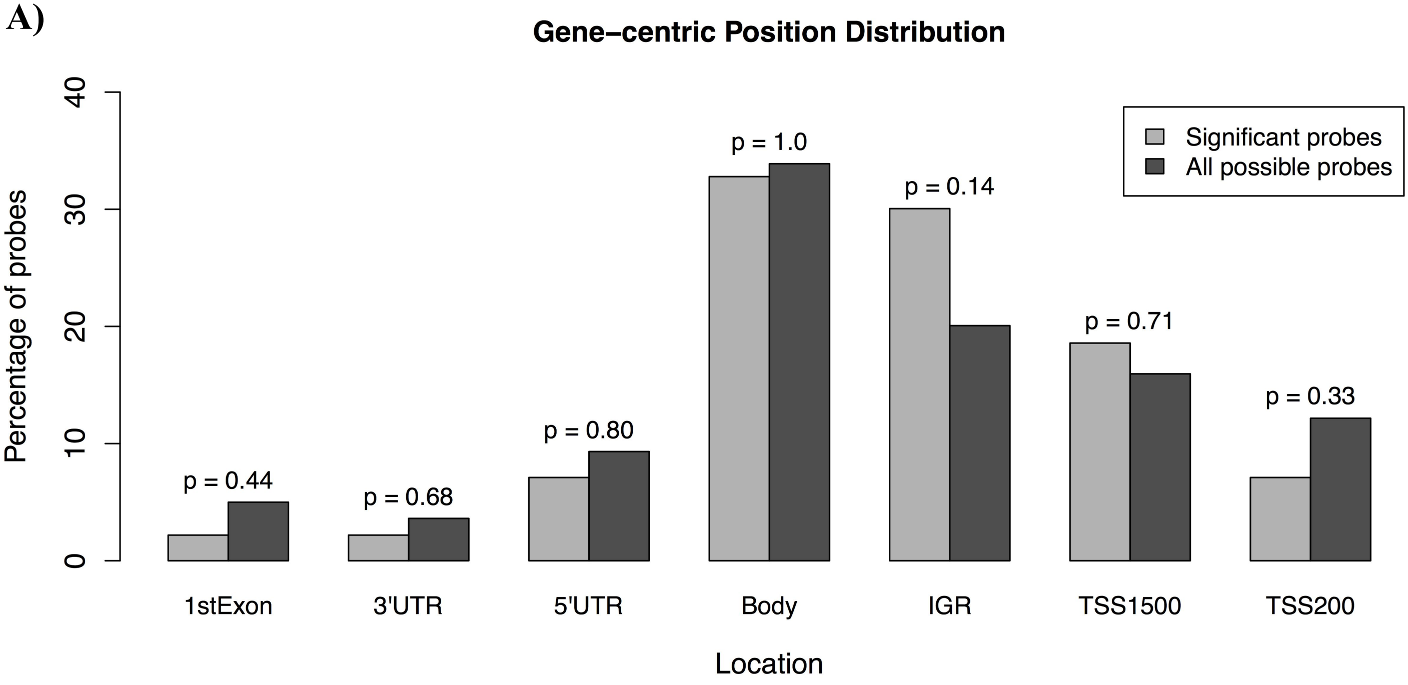

B)

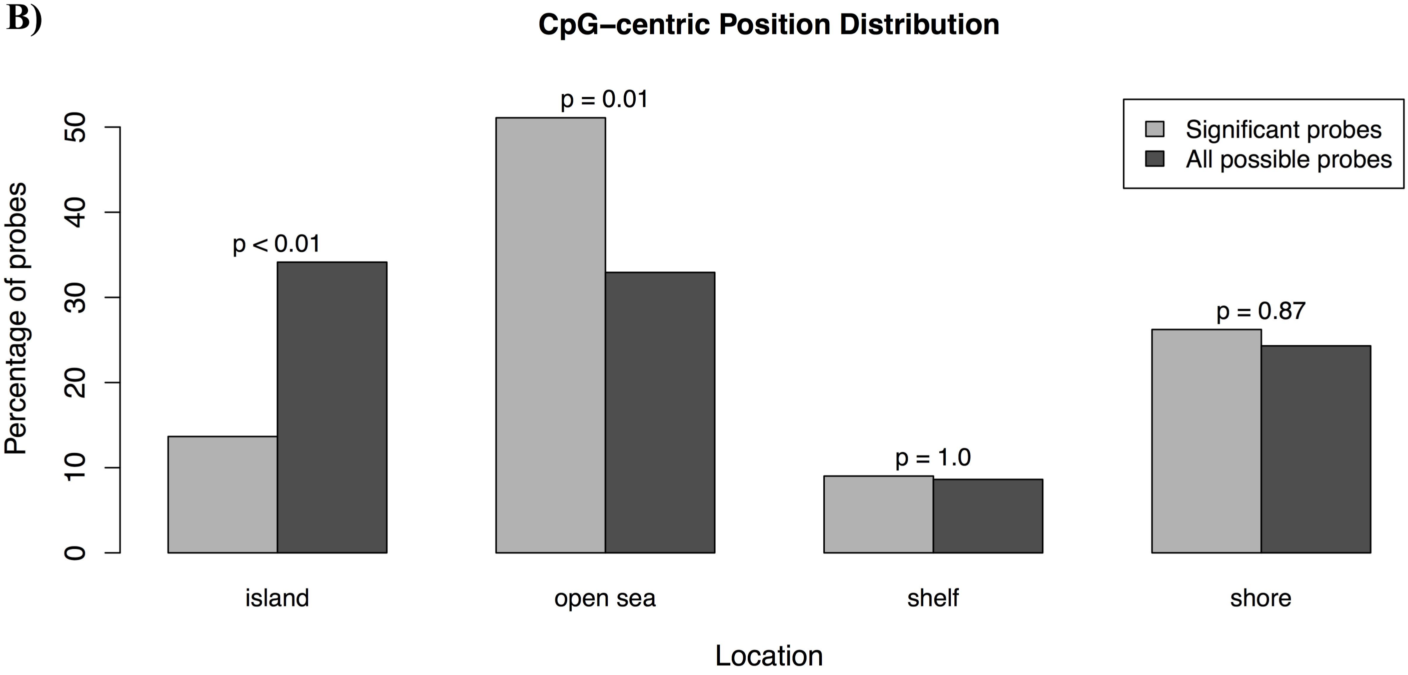

Supplementary Figure 3. Distribution of significantly differentially methylated positions in transcriptional cluster 3 (versus transcriptional cluster 1, corrected for fetal sex and gestational age) (light grey) compared to the full set of possible methylation probes (dark grey), in terms of their (**A**) gene region locations and (**B**) CpG region locations. P-values were obtained from Fisher's exact tests.
