# Supplementary material for: Epigenetic regulation of placental gene expression in transcriptional subtypes of preeclampsia
Source: Clin Epigenetics. 2018 Mar 2;10:28. doi: 10.1186/s13148-018-0463-6 (PMC5833042; doi:10.1186/s13148-018-0463-6)
Supplement: Supplementary file 11 — Figure S4. Remaining functional SMITE modules identified in cluster 2. (PDF 2447 kb) [file 13148_2018_463_MOESM11_ESM.pdf]

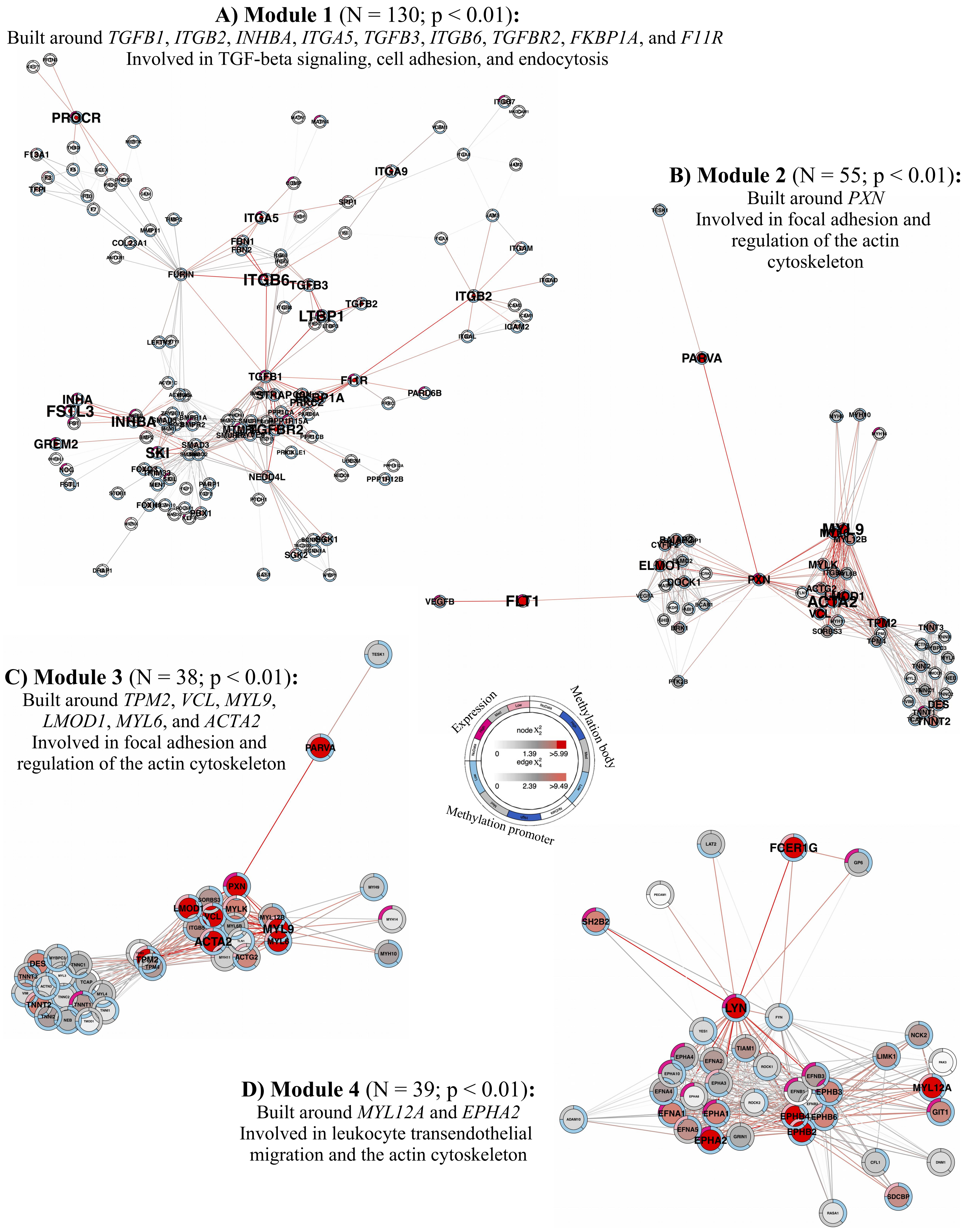
A) Module 1 (N = 130; p < 0.01):
Built around TGFB1, ITGB2, INHBA, ITGA5, TGFB3, ITGB6, TGFBR2, FKBP1A, and F11R
Involved in TGF-beta signaling, cell adhesion, and endocytosis
B) Module 2 (N = 55; p < 0.01):
Built around PXN
Involved in focal adhesion and regulation of the actin cytoskeleton
C) Module 3 (N = 38; p < 0.01):
Built around TPM2, VCL, MYL9, LMOD1, MYL6, and ACTA2
Involved in focal adhesion and regulation of the actin cytoskeleton
D) Module 4 (N = 39; p < 0.01):
Built around MYL12A and EPHA2
Involved in leukocyte transendothelial migration and the actin cytoskeleton
Expression
Methylation body
Methylation promoter
node X2
0
1.39
>5.99
edge X4
0
2.39
>9.49

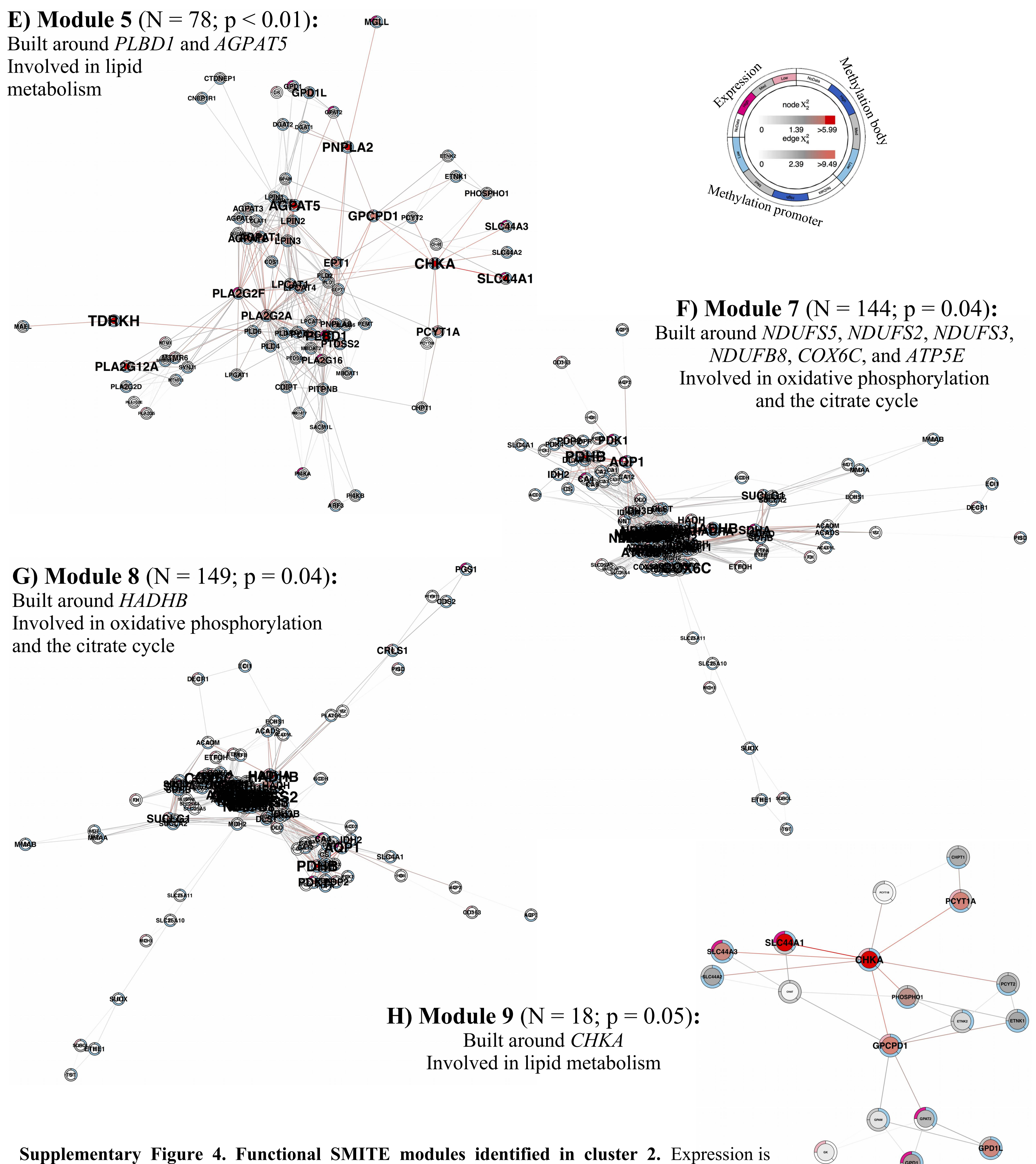

Supplementary Figure 4. Functional SMITE modules identified in cluster 2. Expression is shown on the top left edge of each gene circle (upregulated: dark pink, downregulated: light pink, grey: not significant, white: no data); scored promoter and body methylation are shown on the bottom left and top right of each circle, respectively (hypermethylated: dark blue, hypomethylated: light blue, grey: not significant, white: no data), compared to cluster 1. The symbol text sizes and center node colors are based on the total gene score (low (grey) to high (red)) and the edge colors are representative of the strength of the associations between the genes (low (grey) to high (red)). Cluster 2 module 6 is shown in Figure 3a.
