# Supplementary material for: Epigenetic regulation of placental gene expression in transcriptional subtypes of preeclampsia
Source: Clin Epigenetics. 2018 Mar 2;10:28. doi: 10.1186/s13148-018-0463-6 (PMC5833042; doi:10.1186/s13148-018-0463-6)
Supplement: Supplementary file 14 — Figure S5. Remaining functional SMITE modules identified in cluster 3. (PDF 4125 kb) [file 13148_2018_463_MOESM14_ESM.pdf]

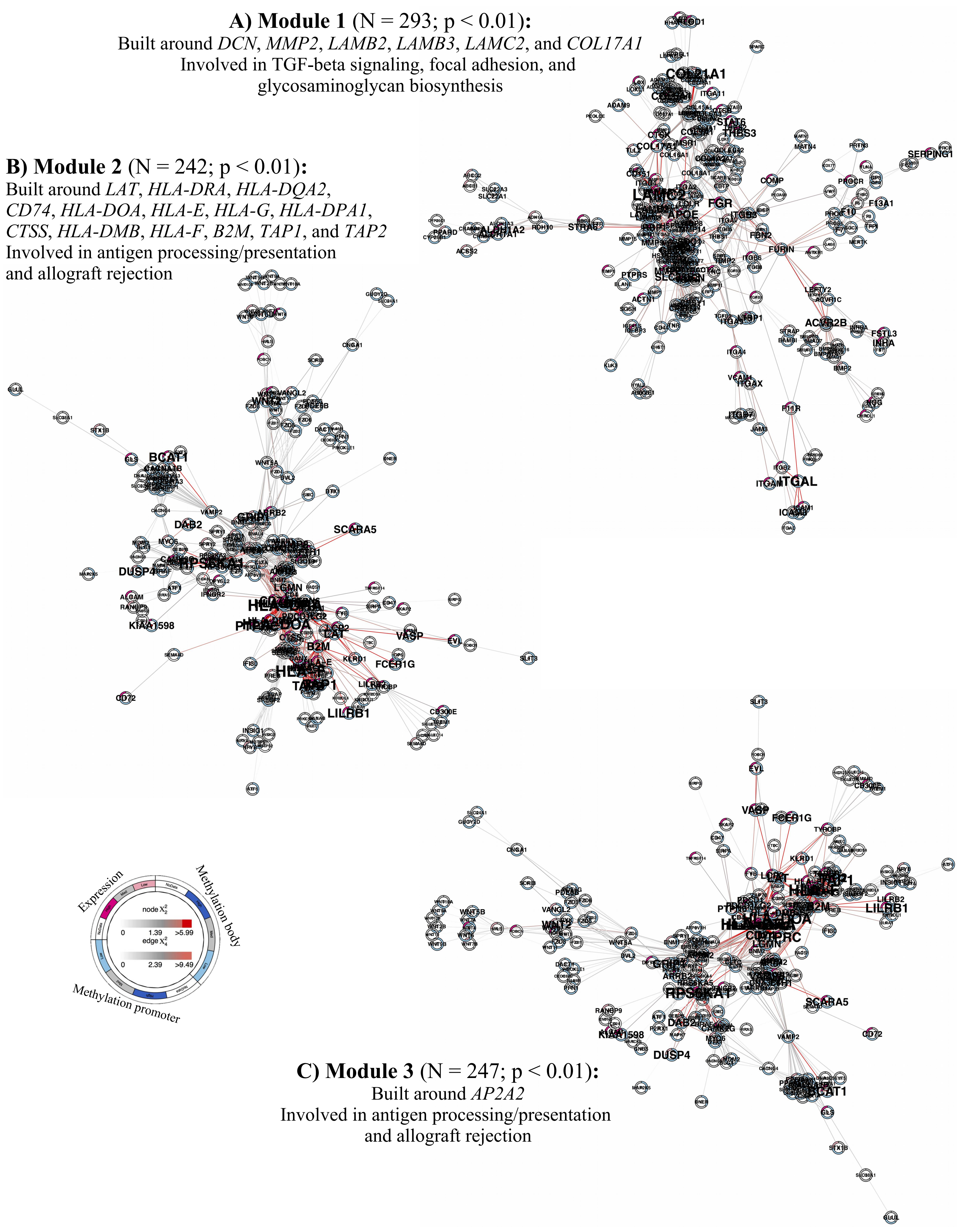
A) Module 1 (N = 293; p < 0.01):
Built around DCN, MMP2, LAMB2, LAMB3, LAMC2, and COL17A1
Involved in TGF-beta signaling, focal adhesion, and glycosaminoglycan biosynthesis
B) Module 2 (N = 242; p < 0.01):
Built around LAT, HLA-DRA, HLA-DQA2, CD74, HLA-DOA, HLA-E, HLA-G, HLA-DPA1, CTSS, HLA-DMB, HLA-F, B2M, TAP1, and TAP2
Involved in antigen processing/presentation and allograft rejection
C) Module 3 (N = 247; p < 0.01):
Built around AP2A2
Involved in antigen processing/presentation and allograft rejection
Expression
Methylation body
Methylation promoter
node X2
0
1.39
>5.99
edge X4
0
2.39
>9.49

D) Module 4 (N = 221; $p < 0.01$)**:**
Built around *SLC35B3*
Involved in TGF-beta signaling, focal adhesion, and glycosaminoglycan biosynthesis

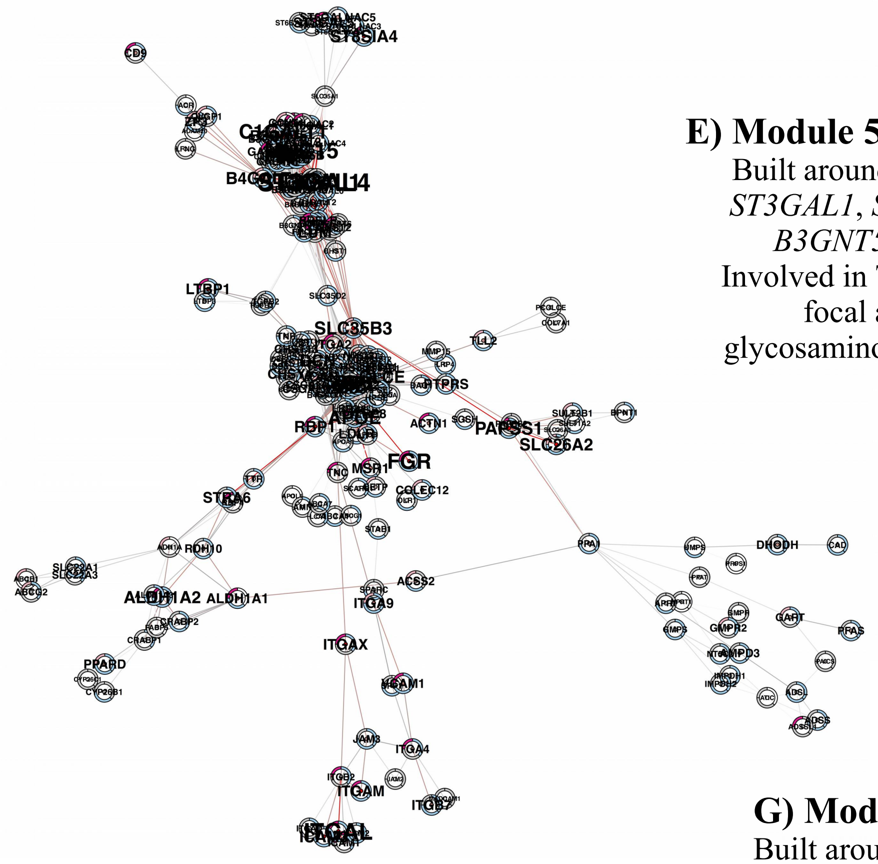

E) Module 5 (N = 65; $p < 0.01$)**:**
Built around *B4GALT1*, *LUM*, *ST3GAL1*, *ST3GAL4*, *MUC15*, *B3GNT5*, and *C1GALT1*
Involved in TGF-beta signaling, focal adhesion, and glycosaminoglycan biosynthesis

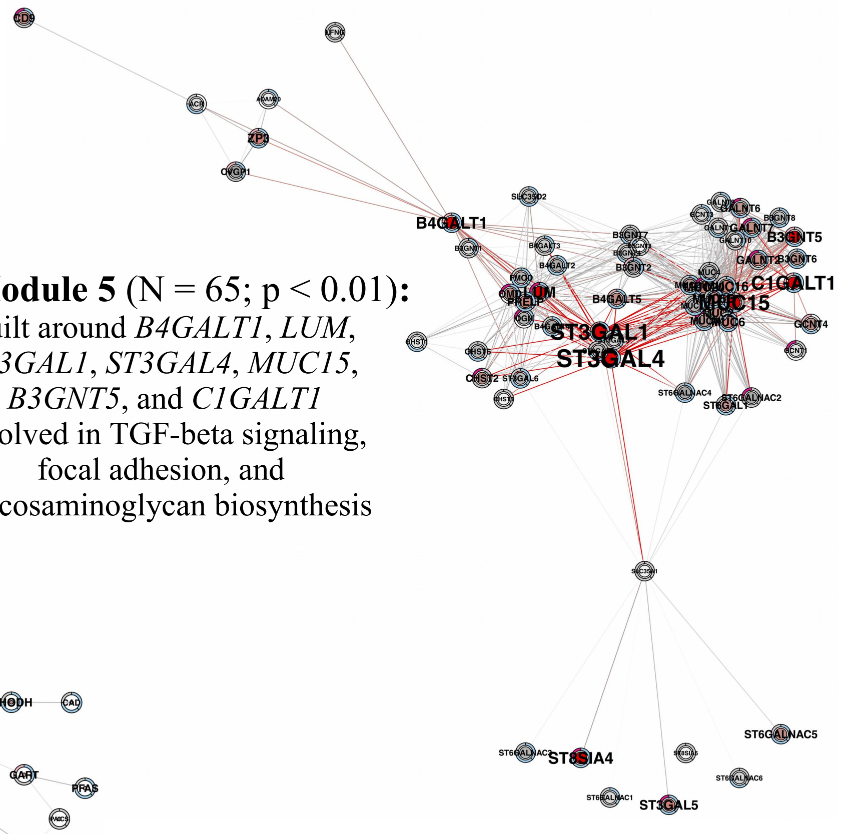

F) Module 6 (N = 162; $p < 0.01$)**:**
Built around *ITGB3*
Involved in TGF-beta signaling, focal adhesion, and glycosaminoglycan biosynthesis

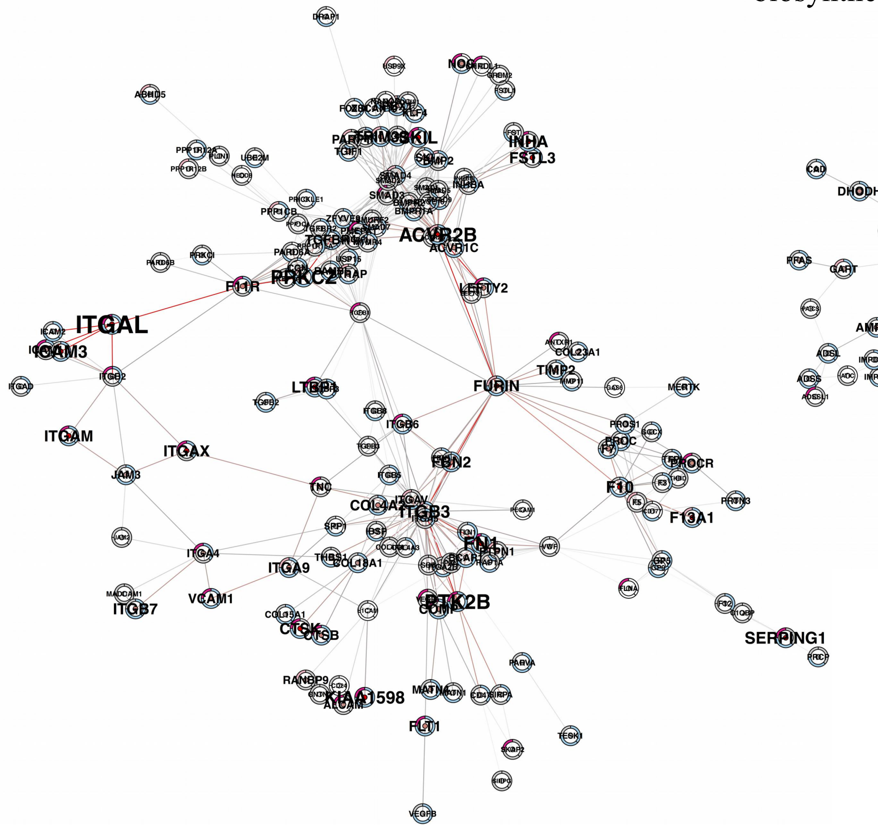

G) Module 7 (N = 157; $p < 0.01$)**:**
Built around *GLCE*, *APOE*, *RBP1*, *ITGAM*, *SDC1*, *ITGAL*, *ITGAX*, *SDC4*, and *ICAM3*
Involved in TGF-beta signaling, focal adhesion, and glycosaminoglycan biosynthesis

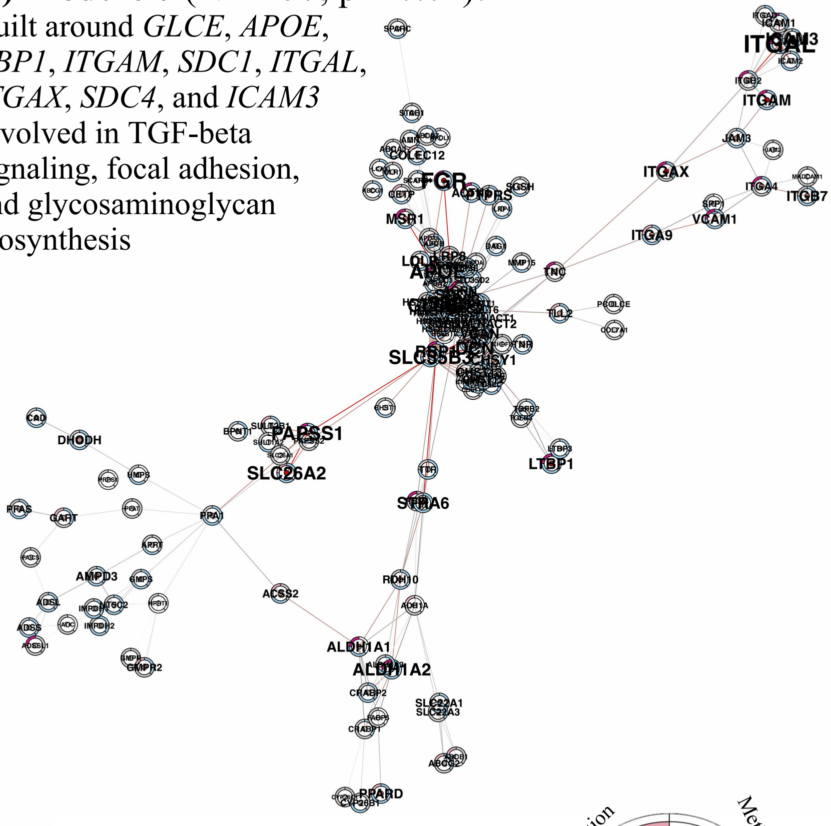

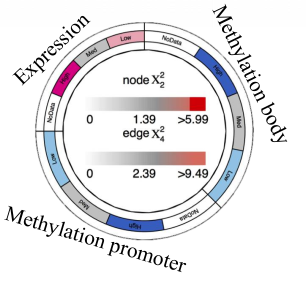

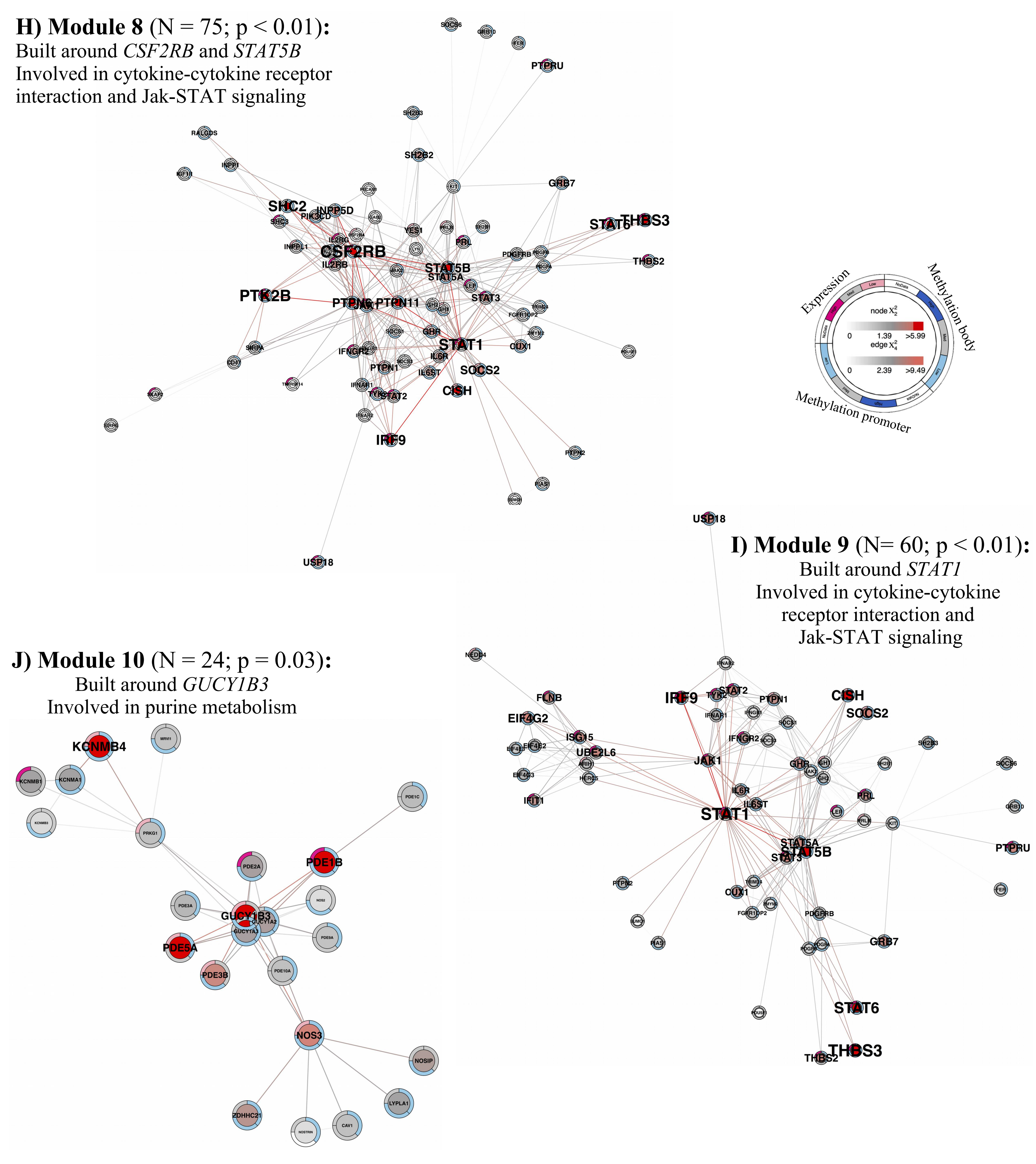

Supplementary Figure 5. Functional SMITE modules identified in cluster 3. Expression is shown on the top left edge of each gene circle (upregulated: dark pink, downregulated: light pink, grey: not significant, white: no data); scored promoter and body methylation are shown on the bottom left and top right of each circle, respectively (hypermethylated: dark blue, hypomethylated: light blue, grey: not significant, white: no data), compared to cluster 1. The symbol text sizes and center node colors are based on the total gene score (low (grey) to high (red)) and the edge colors are representative of the strength of the associations between the genes (low (grey) to high (red)). Cluster 3 module 11 is shown in Figure 3b.
